# Additional value of a combined genetic risk score to standard cardiovascular stratification

**DOI:** 10.1590/1678-4685-GMB-2017-0173

**Published:** 2018

**Authors:** Andreia Pereira, Maria Isabel Mendonca, Sofia Borges, Ana Célia Sousa, Sónia Freitas, Eva Henriques, Mariana Rodrigues, Ana Isabel Freitas, Graça Guerra, Carolina Freitas, Décio Pereira, António Brehm, Roberto Palma Dos Reis

**Affiliations:** ^1^ Unidade de Investigação, Hospital Dr. Nélio Mendonça, Funchal, Portugal; ^2^ Faculdade de Ciências Médicas, Universidade Nova de Lisboa, Lisboa-Portugal; ^3^ Laboratório de Genética Humana, Universidade da Madeira, Campus Universitário da Penteada, Madeira, Portugal

**Keywords:** Coronary artery disease, genetic risk score, Framingham score, risk prediction, risk factors

## Abstract

The utility of genetic risk scores (GRS) as independent risk predictors remains inconclusive. Here, we evaluate the additive value of a multi-locus GRS to the Framingham risk score (FRS) in coronary artery disease (CAD) risk prediction. A total of 2888 individuals (1566 coronary patients and 1322 controls) were divided into three subgroups according to FRS. Multiplicative GRS was determined for 32 genetic variants associated to CAD. Logistic Regression and Area Under the Curve (AUC) were determined first, using the TRF for each FRS subgroup, and secondly, adding GRS. Different models (TRF, TRF+GRS) were used to classify the subjects into risk categories for the FRS 10-year predicted risk. The improvement offered by GRS was expressed as Net Reclassification Index and Integrated Discrimination Improvement. Multivariate analysis showed that GRS was an independent predictor for CAD (OR = 1.87; *p*<0.0001). Diabetes, arterial hypertension, dyslipidemia and smoking status were also independent CAD predictors (*p*<0.05). GRS added predictive value to TRF across all risk subgroups. NRI showed a significant improvement in all categories. In conclusion, GRS provided a better incremental value in intermediate subgroup. In this subgroup, inclusion of genotyping may be considered to better stratify cardiovascular risk.

## Introduction

The most important Traditional Risk factors (TRF) for Coronary Artery Disease (CAD) include dyslipidemia, arterial hypertension, diabetes, obesity, smoking, lack of physical activity and stress (Chan and Boerwinkle, 1994). However, some patients can develop vascular disease without conventional risk factors.

Although the familial nature of CAD has been documented for many years (Marenberg *et al.*, 1994; Lloyd-Jones *et al.*, 2004; Murabito *et al.*, 2005) and the addition of family history has been shown to improve risk prediction (Ridker *et* al., 2007; Ridker *et al.*, 2008), the genetic variants responsible for the increased familial risk were, until recently, unknown. Genome-Wide Association Studies (GWAS) have uncovered several common genetic variants (single nucleotide polymorphisms, or SNPs) that are robustly associated with CAD and have been replicated in multiple independent samples (Welter *et al.*, 2014). The identification of these genetic variants provides an opportunity to evaluate whether addition of a genetic risk score (GRS) to risk models may improve predictive power (Ganna *et al.*, 2013).

There is an increasing interest in the potential use of GRS in cardiovascular disease, because this could increase the number of preventive and therapeutic interventions in individuals and groups with high genetic risk that are not obvious candidates to these interventions using the current standard stratification. Usual cardiovascular risk stratification uses family history, TRF evaluation, and is quantified into scores like Framingham risk score and EuroSCORE (Pencina *et al.*, 2011). However, due to the potential financial and medical costs associated with measuring these new markers like GRS, their ability in improving the prediction of CAD outcomes over existing risk models needs to be rigorously accessed. Effective statistical tools for evaluating the incremental value of the novel markers over the routine clinical risk factors are crucial in the field of outcome prediction.

Here, we evaluate whether a GRS, based on 33 SNPs associated to CAD, is independent of the vascular risk explainable by conventional risk factors and can improve the predictive capacity of TRF in the assessment of CAD risk, according to FRS subgroups (low risk<10%, intermediate risk 10-20% or high risk>20%). Using the Net Reclassification Index (NRI) and Integrated Discrimination Index (IDI), we investigate the performance of combined stratification including TRF and GRS in CAD risk assessment.

## Subjects and Methods

### Study population

A case-control study was performed with 2888 individuals (mean age of 53.0 ± 7.9 years), including 1566 consecutive coronary patients and 1322 controls. These were divided into three subgroups according to FRS: low risk (FRS<10, n = 1312, 68.7% male), intermediate risk (10≤FRS≤20, n = 1049, 83.1% male) and high risk (FRS>20, n = 527, 90.1% male). Cases and controls were matched for age and gender.

Determination of CAD was adjucated by a dedicated intervention cardiologist. Angiographically proven CAD was considered significant if ≥1 coronary lesions of ≥70% stenosis in ≥1 major coronary artery or its primary branches. Absent or non-flow limiting CAD was excluded from this study. The control group consisted of healthy volunteers selected from the same population with no symptoms or history of CAD. All controls underwent clinical and phenotype assessment of TRF, an electrocardiogram, and, if needed, complementary exercises stress tests or a Stress Echocardiography. Population stratification analysis was performed in our population set to account for possible genetic admixture and no significant genetic outliers (<5%) were identified with Principal Component Analysis (PCA) (Abdi and Williams, 2010).

The study was conducted according to the Declaration of Helsinki, the protocol was reviewed and approved by the Hospital ethics committee, and all patients provided written informed consent.

### Data collection

Data was collected from all subjects in a standardized file comprising demographic, clinical characteristics and TRF (gender, age, level of exercise, smoking status, arterial hypertension, dyslipidemia, diabetes, and family history of CAD, body mass index (BMI), heart rate and pulse wave velocity (PWV). ‘Smoking status’ refers to current smokers or subjects with less than 5 years of smoking cessation (Mons *et al.*, 2015).

Arterial Hypertension was considered when patients, at the entry into this study, were already diagnosed and/or had been on antihypertensive medication for more than 3 months, or newly diagnosed hypertensives with systolic blood pressure (SBP)/diastolic blood pressure (DBP) ≥140/90 mmHg measured on at least 3 occasions (Chobanian *et al.*, 2003).

Subjects with LDL>100 mg/dL, HDL<40 mg/dL for men, and<45 mg/dL for women, non HDL (Total cholesterol – HDL)>130 mg/dL, triglycerides>150 mg/dL or Apo B>100 mg/dL were classified as having dyslipidemia (Expert Panel on Detection Evaluation and Treatment of High Blood Cholesterol in adults, 2001). Subjects were classified as having diabetes if taking oral anti-diabetic medication or insulin or if fasting plasma glucose was higher than 7.0 mmol/l or 126 mg/dL (Genuth *et al.*, 2003).

Family history of CAD was considered if a female relative (mother or sister) had presumed CAD disease before 65 years, or a male relative (father or brother) had CAD disease before 55 years old. The definition of other traditional risk factors was based on standard criteria, as previously reported (Asmar *et al.*, 1995; National Institute of Health, National Heart, Lung, and Blood Institute North American Association for the Study of Obesity, 2000).

### Biochemical analysis

Blood samples were extracted after 14-16 hours fasting. Biochemical analyses were performed at the Central Laboratory of the Hospital, according to standard techniques. For measurement of total cholesterol, HDL, LDL, triglycerides and glucose, blood samples were placed in dry tubes, centrifuged half an hour late at 3500 *xg* and subsequently quantified by an enzymatic technique using an AU 5400 autoanalyzer (Beckman Coulter). Biochemical markers such as lipoprotein-a (Lp(a)), apolipoprotein B (Apo B), and high sensitivity C-Reactive Protein (hs-CRP) were quantified by Immunoturbidimetry using an AU 5400 automatic system (Beckman Coulter).

### SNP selection

SNPs were selected either from GWAS or candidate gene association studies (Coronary Artery Disease Consortium, 2011; Schunkert *et al.*, 2011). These SNPs were either previously tested in a sample of our population, or in a genetically similar southern European cohort. Entering criteria included genes described in previous studies with an Odds Ratio (OR) for CAD ≥ 1.1 and, simultaneously, with a Minor Allele Frequency (MAF)>5%. Genes with low Hardy-Weinberg equilibrium p<0.002 (after Bonferroni correction) were excluded.

According to their possible CAD-related function, we have included 32 genes associated to cell cycle, cellular migration and inflammation (rs1333049 (9p21.3), rs4977574 (CDKN2B), rs618675 (GJA4), rs17228212 (SMAD3), rs17465637 (MIA3), rs12190287 (TCF21), rs3825807 (ADAMTS7), rs11556924 (ZC3HC1), rs12526453 (PHACTR1); genes involved in pro-oxidative status (rs1801133 (MTHFR 677), rs1801131 (MTHFR 1298), rs705379 (PON 1), rs662 (PON192), rs854560 (PON 55), rs6922269 (MTHFD1L); genes associated with modifiable risk factors such as lipids metabolism, hypertension and diabetes/obesity (rs2114580 (PCSK9), rs20455 (KIF6), rs7412/rs429358 (APOE), rs964184 (ZNF259), rs599839 (PSRC1), rs5186 (AT1R), rs699 (AGT), rs4340 (ACE), rs4402960 (IGF2BP2), rs1326634 (SLC30A8), rs266729 (ADIPOQ), rs7903146 (TCF7L2), rs17782313 (MC4R), rs1801282 (PPARG), rs1884613 (HNF4A), rs8050136 (FTO) and rs1376251 (TAS2R 50).

Further SNP inclusion was not performed at this time due to low expectation of improvement in overall risk reclassification and cost effectiveness limitations.

Details of SNPs used in GRS model are presented in Supplementary Table S1.

### Genotype analysis

Genetic analysis was performed at the Human Genetics Laboratory of the University of Madeira. Genomic DNA was extracted from 80 μL of peripheral blood using a standard phenol-chloroform method. A TaqMan allelic discrimination assay for genotyping was performed using labeled probes and primers pre-established by the supplier (TaqMan SNP Genotyping Assays, Applied Biosystems). All reactions were done on an Applied Biosystems 7300 Real Time PCR System and genotypes were determined using the 7300 System SDS Software (Applied Biosystems) without any prior knowledge of the individual’s clinical data. Quality check of genotyping techniques was maintained by the inclusion of one non-template control (NTC) in each plate of 96 wells. All SNPs TaqMan assays had blind duplicates accounting for 20% of all samples. Some SNP genotypes were randomly confirmed by conventional direct DNA sequencing, as 10-15% of all samples were re-amplified for sequencing.

### Statistical analysis

Deviation from Hardy-Weinberg equilibrium for the 33 genotypes at individual loci were assessed using the Chi-square test and *p*<0.002 with Bonferroni correction for all SNPs included. LPA gene variant was excluded for further analyses due to its low Hardy-Weinberg equilibrium (*p*<0.002).

Comparisons of baseline characteristics and biochemical data of cases and controls were analyzed by Chi-square tests for categorical variables, and Student’s *t*-test or Mann- Whitney tests were performed for continuous variables, as appropriate. Genotypic frequencies were determined from observed counts and compared by Chi-square analysis.

To evaluate the impact of genotype frequencies and Odds Ratios (OR) on the overall discriminative accuracy of genetic risk models, we assessed the AUC. SNPs associated with *p*-values less than or equal to 0.05 entered the race-specific GRS with a coding value of 2 for the mutated genotype (risk), 1 for the heterozygote, and 0 for the homozygous wild-type genotype. The GRS was constructed under a multiplicative model (multiplying the odds ratio of each genotype for all the 32 genetic variants). The maximum number of risk alleles possessed by one individual was 36 and the minimum was 13 (mean ± standard deviation (SD) = 23.96 ± 3.63). The increasing loci were assumed to have an additive effect, and the GRS was normally distributed.

**Figure 1:**
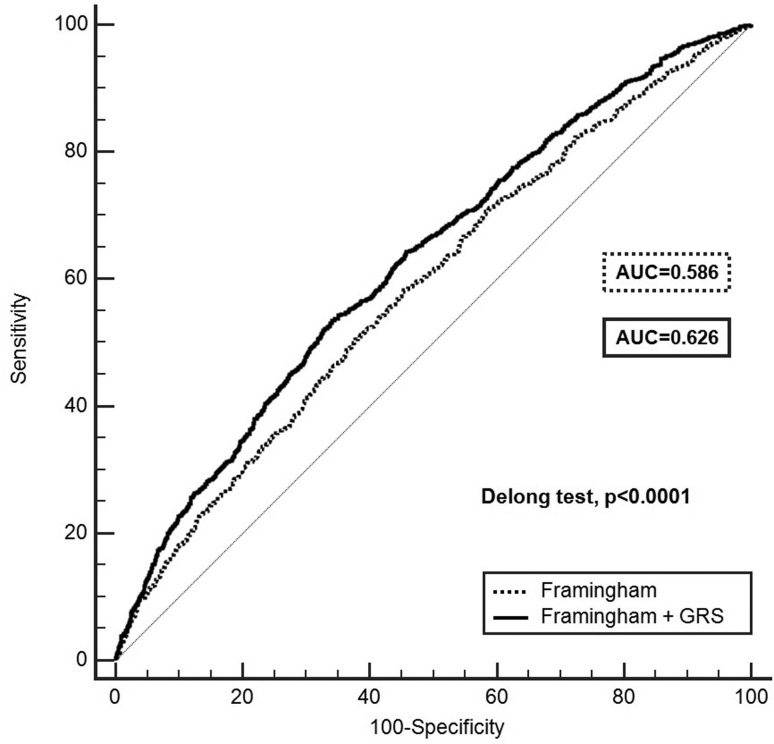
ROC curves based on the GRS + Framingham model compard to Framingham model alone. The two curves are based on logistic regression models incorporating Framingham risk with and without GRS. AUC indicates area under curve. The DeLong test compares the difference between the two AUCs and showed statistical significance.

ROC curves based on the GRS + Framingham model were compared to a Framingham model alone (Figure 1). For each risk subgroup, the GRS was included in a ROC model containing the TRF. A new combined risk score was calculated for each individual (TRF + GRS) by multiplying each model coefficient by the associated risk variable and then summing the products. The AUC equals the probability that a classifier will rank a randomly chosen positive instance higher than a randomly chosen negative one. ROC curve analysis was performed using the MedCalc software. Non-parametric methods developed by DeLong *et al.* (1988) were used to test for significant differences between ROC curves.

NRI was computed according to the continuous method and applied to the case- control studies (Pencina *et al.*, 2011). We further calculated categorical NRI for each Framingham subgroup. In each risk group the number of individuals reclassified intohigher and lower risk categories is presented (Supplementary Table S2). NRI was defined as the percentage of subjects changing categories in each subgroup when adding the new markers and IDI as the improvement of the difference in average of predicted probabilities between cases and controls. Both measures were obtained with the PredictABEL package available in R software (version 3.2.0). Discriminative capacity (AUC) was accessed for evaluation of specificity and sensitivity with MedCalc version 13.3.3.0, and other statistics were analyzed by the Statistical Package for the Social Sciences, SAS Software version 19.0). All *p*-values were two-sided, and statistically significant for *p*<0.05.

## Results

Table 1 compares baseline characteristics between CAD and control groups. According to the selection criteria, age and gender did not differ significantly between the two groups. In contrast, CAD patients were more likely to have a higher prevalence of modifiable risk factors, such as dyslipidemia, tobacco use, hypertension, and diabetes (*p*<0.05). Also BMI, PWV, family history of CAD and lower level of exercise were significantly more frequent among cases (*p*<0.05). Lower heart rates among patients potentially reflect a better heart rate control (*p*<0.0001). Across all Framingham score subgroups, the GRS was significantly lower in controls that CAD patients (*p*<0.0001).

**Table 1:** Baseline characteristics for both CAD patients and controls.

Variables	Total	Cases	Controls	*p*-value
	(n = 2888)	(n = 1566)	(n = 1322)	
Male sex, *n* (%)	2248 (77.8)	1238 (79.1)	1010 (76.4)	0.087
Age, years	53 ± 7.9	53.3 ± 8	52.7 ± 7.8	0.053
Dyslipidemia^†^, *n* (%)	2344 (81.2)	1414 (90.3)	930 (70.3)	<0.0001
Total Cholesterol, mg/dl	193 (77-437)	180 (77-437)	205 (92-360)	<0.0001
HDL, mg/dl	44 (12-116)	41 (18.2-115.8)	48 (12-116)	<0.0001
LDL, mg/dl	114.8 (9.6-298)	104.6 (15.6-298)	127.2 (9.6-251)	<0.0001
Lipoprotein (a), mg/dl	15.1 (0.5-241)	20.4 (0.5-241)	12.8 (0.6-236)	<0.0001
Apolipoprotein B, mg/dl	93.2 (2.9-256.9)	93.9 (4.9-256.9)	92.5 (2.9-212.7)	<0.0001
Triglycerides, mg/dl	132 (30-2500)	141 (31-2500)	121 (30-1361)	<0.0001
Smoking status^•^, *n* (%)	1039 (36)	730 (46.6)	309 (23.4)	<0.0001
Hypertension, *n* (%)	1814 (62.8)	1114 (71.1)	700 (53)	<0.0001
SBP, mmHg	137.1 ± 19.6	137.9 ± 20.8	136.2 ± 18.1	0.024
DBP, mmHg	83.2 ± 11.5	82.6 ± 11.8	83.9 ± 11.1	0.002
PWV, m/s	8.5 ± 1.9	8.6 ± 1.9	8.3 ± 1.7	<0.0001
Diabetes, *n* (%)	708 (24.5)	533 (34)	175 (13.2)	<0.0001
Fasting glucose, mg/dl	102 (53-458)	106 (53-458)	99 (71-393)	<0.0001
BMI, kg/m^2^	28.4 ± 4.4	28.6 ± 4.2	28.1 ± 4.5	0.007
Level of exercise^#^, *n* (%)	1334 (46.2)	573 (36.6)	761 (57.6)	<0.0001
Family history of CAD, *n* (%)	540 (18.7)	373 (23.8)	167 (12.6)	<0.0001
Heart rate, bpm	70.4 ± 12.2	68.8 ± 12.5	72.3 ± 11.5	<0.0001
FRS<10, *n* (%)	1312 (45.4)	629 (40.2)	683 (51.7)	^*^
GRS	0.58 ± 0.7	0.69 ± 0.8	0.49 ± 0.6	<0.0001
10≤ FRS≤ 20, *n* (%)	1049 (36.3)	583 (37.2)	466 (35.2)	^*^
GRS	0.56 ± 0.6	0.62 ± 0.7	0.48 ± 0.4	<0.0001
FRS>20, *n* (%)	527 (18.2)	354 (22.6)	173 (13.1)	^*^
GRS	0.62 ± 0.6	0.70 ± 0.7	0.47 ± 0.4	<0.0001

† LDL>100, HDL<40 for men and <45 for women, Triglycerides>150 and Apo B>100; HDL, High-density lipoprotein; LDL, Low-density lipoprotein;

• Current smokers or<5 years of cessation; SBP, Systolic blood pressure; DBP, Diastolic blood pressure; PWV, Pulse wave velocity; BMI, Body mass index;

# More than 40min/week; CAD, Coronary Artery Disease; bpm, beat per minute; FRS – 10-year Framingham risk score in %

* Comparison of the 3 subgroups of FRS, *p*<0.0001; GRS, Genetic risk score; Biochemical variables are presented by median (minimum - maximum) and other continuous variables with mean ± standard deviation. Statistically significant for *p*<0.05.

A logistic regression under a Forward Wald method was performed including GRS and TRF (Table 2). In this multivariable analysis, GRS was an independent predictor for CAD (OR = 1.87; 95%CI: 1.58 – 2.21), with statistical significance (p<0.0001). Even after a Bonferroni Correction, GRS remained significant at a *p*<0.01 (0.05/5) level. Furthermore, smoking status (OR = 3.44; 95%CI: 2.89 – 4.10); diabetes (OR = 3.19; 95% CI: 2.61 – 3.91); arterial hypertension (OR = 2.10; 95% CI: 1.77 – 2.48) and dyslipidemia (OR = 1.30; 95% CI: 1.03 – 1.65) were independently and significantly associated to CAD risk (*p*<0.05) (Table 2).

**Table 2:** Logistic Regression^*^model for CAD in subgroups of Framingham 10-year risk (< 10; 10-20%;>20%) and in total population.

Variables	FRS<10%		FRS 10%-20%		FRS>20%		Total	
	OR	*p*-value	OR	*p*-value	OR	*p*-value	OR	*p*-value
	(95% CI)		(95% CI)		(95% CI)		(95% CI)	
Hypertension	3.25 (2.52 - 4.18)	<0.0001	1.88 (1.39 - 2.56)	<0.0001	---	---	2.10 (1.77 - 2.48)	<0.0001
Diabetes	2.93 (1.90 - 4.52)	<0.0001	4.34 (3.14 - 6)	<0.0001	3.56 (2.34 - 5.4)	<0.0001	3.19 (2.61 - 3.91)	<0.0001
Dyslipidemia	1.71 (1.28 - 2.29)	<0.0001	---	---	---	---	1.30 (1.03 - 1.65)	0.030
Smoking status	4.92 (3.66 - 6.62)	<0.0001	3.57 (2.65 - 4.8)	<0.0001	3.11 (2.04 - 4.73)	<0.0001	3.44 (2.89 - 4.1)	<0.0001
GRS	1.76 (1.41 - 2.2)	<0.0001	1.86 (1.39 - 2.49)	<0.0001	2.49 (1.54 - 4.05)	<0.0001	1.87 (1.58 - 2.21)	<0.0001
Constant	0.16	<0.0001	0.24	<0.0001	0.342	<0.0001	0.21	<0.0001

* Using Forward Wald Conditional Regression method (SPSS v. 19.0). FRS, Framingham risk score; OR, Odds ratio; CI, Confidence interval; GRS, Genetic risk score; Dashed points represent co-variables not significant after adjusted multivariate analysis; Statistically significant for *p*<0.05.

We compared the AUC for the Framingham + GRS model with the basal AUC for the Framingham model, as shown in Figure 1. Framingham yielded an AUC of 0.586 (95% CI: 0.568 – 0.605), predicting CAD moderately well. The model including the GRS was more discriminative than Framingham alone (AUC = 0.626; 95% CI: 0.608 – 0.644; DeLong test p<0.0001). The increase in predictive accuracy for the Framingham + GRS model was 4% (95% CI: 2.6% - 5.4%).

For each subgroup of Framingham, we found that including the GRS to the model with TRF only, the AUC increased significantly (Table 3), showing that GRS adds predictive value to TRF in all the 3 risk subgroups. Specifically, in individuals within the low risk subgroup, the AUC was 0.72 for the TRF model and 0.75 for the TRF + GRS model (*p*<0.0001). The increase in predictive accuracy in this subgroup was 2.5% (95% CI: 1.4 – 3.6%). This difference was small, but significant (DeLong test for correlated ROC curves *p*<0.0001). Similarly, in individuals within intermediate FRS subgroup, the AUC was 0.70 for the TRF model and 0.73 for the TRF + GRS model. The increase in the predictive accuracy for the AUC model in this subgroup was 2.6% (95% CI: 1.2 – 3.9%) at a significant p level (*p* = 0.0002). Finally, a surprising significant (DeLong’s test for correlated ROC curves p = 0.003) addition to the model’s predictive accuracy was seen in the higher risk of FRS group with an AUC increasing from 0.68 to 0.72. The increase in predictive accuracy for the AUC model (TRF + GRS model) in this group was 4.4% (95% CI: 1.5 – 7.3%). For all subgroups, the 95% CI of the increase did not include zero, suggesting that the increase in AUC caused by the GRS inclusion is statistically significant. The addition of GRS to TRF improved the risk classification of the models. The new marker provided a continuous NRI of 32.3% (95%CI: 22.4-42.3%; *p*<0.0001) in the low risk group; 30.4% (95%CI: 19.0-41.8%; *p*<0.0001) in the intermediate FRS group and 29.8% (95%CI: 13.1-46.6%; *p* = 0.0005) in the high risk subgroup (Table 3). Furthermore, the inclusion of GRS to TRF also provided an IDI of 2.4% (95%CI: 1.6-3.2%; *p*<0.0001) in the low risk group; 2.1% (95%CI: 1.3-2.9%; *p*<0.0001) in the intermediate risk group and 3.3% (95%CI: 1.9-4.7%; *p*<0.0001) in the high-risk group. Full reclassification results are provided in Table 3.

**Table 3:** Reclassification table comparing predicted CAD risk with and without GRS.

		FRS		
	Total	<10%	10-20%	>20%
	(n = 2888)	(n = 1312)	(n = 1049)	(n = 527)
CAD patients (n)	1566	629	583	354
Controls (n)	1322	683	466	173
NRI (%)	31.7	32.3	30.4	29.8
(25.0-38.4)	(22.4-42.3)	(19.0-41.8)	(13.1-46.6)	
(95% CI)	*p*<0.0001	*p*<0.0001	*p*<0.0001	*p* = 0.0005
IDI (%)	2.3	2.4	2.1	3.3
(95% CI)	(1.8-2.9)	(1.6-3.2)	(1.3-2.9)	(1.9-4.7)
	*p*<0.0001	*p*<0.0001	*p*<0.0001	*p*<0.0001
AUC				
TRF	0.716	0.724	0.701	0.676
(95% CI)	(0.699-0.732)	(0.699-0.748)	(0.672-0.728)	(0.634-0.716)
TRF+GRS	0.741	0.749	0.726	0.720
(95% CI)	(0.724-0.757)	(0.725-0.772)	(0.698-0.753)	(0.680-0.758)
*p-*value for the difference	<0.0001	0.0001	0.0002	0.003
Nagelkerke R Square				
TRF	0.190	0.213	0.166	0.138
TRF+GRS	0.219	0.242	0.191	0.184

FRS, 10-year Framingham risk score; CAD, Coronary Artery Disease; NRI, Net Reclassification Improvement continuous NRI); CI, Confidence Interval; IDI, Integrated Discrimination Improvement; AUC, Area Under the receiver operating characteristic Curve; TRF, Traditional Risk Factors (Dyslipidemia, Smoking, Diabetes and Hypertension); GRS, Genetic risk score.

The Nagelkerke R square value increased in each FRS subgroup with inclusion of GRS, that is, this new variable improved the proportion of variation explained in the model. Therefore, it can be stated that the disease is explained at 19.1% by the independent variables in the intermediate risk group. The values presented in Table 3 confirm that the selected variables present a significant explanatory degree about the dependent variable.

A complementary study of categorical NRI is shown in Supplementary Table S2. In the Framingham low risk subgroup (< 10%), a 27.5% NRI was found in the control population and a -7.8% NRI in CAD subgroup. In intermediate Framingham risk subgroup (10-20%), the opposite result was found with 18% of CAD patients being reclassified into higher risk whereas 0.6% of controls into lower risk. Finally, for the higher risk subgroup (> 20%), both patients and controls were reclassified into higher risk groups (5.9% and - 1.7%, respectively). Overall reclassification for each Framingham subgroup (case- control) was positive and expressed as a categorical NRI of 19.7%, 18.7% and 4.2%, respectively.

## Discussion

We have downgraded our expectations after an unfulfilled enthusiastic time of expecting genes to account for hidden heritability in CAD, not meant for generalized use in the actual guidelines for cardiovascular prevention. For 1-5% of allelic frequencies and ORs for CAD/MI at GWAS level from 1.1- 1.5 reported in the literature will produce moderate risk improvement if added to standard risk stratification models, such as Framingham (Morrison *et al.*, 2007; Van der Net *et al.*, 2009). These models have reasonable to good risk prediction curves, as modeled with C-statistics ranging from 68-72% (Humphries *et al.*, 2008; Paynter *et al.*, 2010). Nevertheless improved accuracy is expected when gene-gene inter-mapping and the impact of some epigenetic factors are accounted for in CAD risk.

The current case-control study assesses the contribution of genetic risk for CAD in a southern European population controlled for age and sex. In the multivariate model, GRS presents a cardiovascular risk independent of the risk conferred by TRF. Thus, it can improve the predictive capacity of TRF scores. According to the results, standard risk Framingham evaluation states good AUC levels across all risk subgroups, with an AUC ranging from 0.676 to 0.749. Apart from its mathematical calculation, the intermediate risk subgroup represents the vast majority of patients followed at the outpatient clinics. The low-age patient with one or two risk factors and low calculated cardiovascular risk is frequently left out of intensive prevention, such as statin introduction, active measures for tobacco eviction, or inclusion in exercise program.

Here, we report that a GRS based on 32 CAD risk alleles, identified previously in GWAS databases, independently and significantly predicts CAD with an increased risk of 80% (1.8 times). Our observations line up with several reports already addressing the impact of adding GRS to standard risk stratification. Recently, Iribarren *et al.* (2016) have reported, in a large scale cohort living in the US, improved predictive capacity and discrimination index for four GRS starting from just 8 or 12 and up to 36 to 51 genetic variants. Likewise Tada *et al.* (2016) also reported that in a panel of 23,595 participants followed-up for 14 years, two GRS of 27 and 50 SNPs improved all measures of discrimination and reclassification.

Despite the modest predictive ability, our genetic risk model seems useful in preventive health care and disease prevention to correctly identify individuals at intermediate and high-risk groups. We observed a general tendency for both measures of reclassification improvement, the NRI and IDI, to increase after addition of the GRS to the basic risk function. The continuous NRI was 30.8% for the entire cohort (95% CI: 24.0%-37.5%). However, reclassification improvement was more noticeable in the intermediate risk group, where it was statistically significant (NRI: 33.7%, 95%CI 25.6%-41.8%; IDI: 1.8%, 95%CI 1.2%-2.3%). It is important to note that the constructed reclassification tables follow clinically relevant strata of 5, and 20 percent 10-year risk, in order to have direct clinical application. But limitations in the interpretation of data apply, because the prevalence of CAD in any patient study population is much higher than in a normal population, and thus, frequencies and risks are highly overestimated. The feasibility and clinical application of the GRS approach strategy will depend not only on the predictive capacity of the risk model, but also on the threshold level that is chosen.

We believe that genetic information will improve preventive strategies targeting individuals at very high genetic risk even though this may be a small subgroup. Nevertheless, an issue that deserves remarks relates to genetic information, adverse events, and even cardiovascular mortality. It is irrefutable that GRS is associated with worse prognosis and outcomes. Several GRS, including SNPs associated with CHD, have been associated with incident events in distinct cohorts (CARDIoGRAMplusC4d Consortium, 2013).

These studies have been conducted in both primary and secondary prevention subpopulations. Long-term prospective cohorts have validated GRS as a marker for incident events across all risk categories in primary care (Voight *et al.*, 2012; Krarup *et al.*, 2015). Mega *et al.* (2015) have also analyzed GRS orientated therapy with statins reporting interesting number needed to treat reaching just 20 to 25 patients with high GRS to avoid one incident adverse outcome. Also, Voight *et al.* (2012) have already tested GRS in a Mendelian randomization design trial, and thus, GRS has been pointed out as an effective tool for early statin introduction in patients with a concordant genotypic score and phenotypic LDL elevation (Voight *et al.*, 2012). Similarly, in a secondary prevention setting, our group has reported, in 1555 patients with documented CAD, significantly higher cardiovascular mortality in patients with higher GRS (Pereira *et al.*, 2017).

Considerably disparate numbers of gene variants and different population settings have been validated in these trials, ranging from 13 to a few hundred genes. Not in all of these studies with positive association with adverse outcomes at the long-term follow-up, the NRI was statistically significant (Ripatti *et al.*, 2010). But this was not a justification for its clinical uses (Pencina *et al.*, 2009). Furthermore, Pencina *et al.* (2011) have already addressed this limitation of comparing NRI, stating that continuous NRI may not be adequate in case-control studies. Similarly, distinct risk cut-offs and the number of risk categories in categorical NRI make inter-study comparisons rather difficult. Nevertheless, and apart from the limitation of using NRI to improve models that already perform well (Pencina *et al.*, 2011), in this study, both continuous and categorical metric formulas can be of sufficient discrimination power to be useful in clinical settings, especially regarding the intermediate risk group, where classical and standard risk stratification seems to be open to sampling errors.

Irrespective of their role in preventive strategy control and relative mortality reduction, classical risk factors can change over time with lifestyle and drug interventions. Also, many biochemical and phenotypic biomarkers, like hs-CRP and PWV, are powerful predictors of disease risk for short-term concern, but less accurate in assessing lifetime risk. Screening for risk stratification is an important issue. Genetic information is an attractive measure for risk prediction with a number of advantages over classical risk stratification. Though its true predictive power in different clinical scenarios can be quite disparate, by being self-tailored and highly stable over time its use may gain preference over classical measures. Furthermore, as It is expected that financial cost related to genome sequencing will further decline in the near future and, already in short-term, additional genetic prediction will become available as regular biomarker determinations and, in this way, can reach larger population coverage. Our findings confirm the importance of genetic contributions to CAD disease even when adjusting for traditional risk stratification.

### Strengths and limitations of the study

A significant strength was the use of a genetically isolated population. This has been especially valuable for mapping rare recessive disorders, but many researchers believe that this could be a solution for more complex disorders as well, because of the relatively uniform genetic background of the population. Some culturally and genetically isolated populations have a more similar lifestyle and share eating habits and a natural environment, thus reducing environmental variation. Often, these populations have been founded by a small number of individuals, followed by a period of genetic isolation, during which genetic drift might have been seen, and population expansion mainly occurred by population growth and not by immigration (Jorde and Wooding, 2004). Another strength of this study is the use of a gold standard of angiography for CAD phenotype assignment, which refines the group of interest.

Our study has included a limited number of genetic variants associated with CAD. Although there is no established minimal number of genes to include in a genetic score, and this number may depend on allele frequencies specific to the population under analysis, the inclusion of more SNPs into our score could increase the predictive power for CAD and other outcomes. Further studies evaluating the clinical utility of adding a GRS in very large samples of individuals are underway.

Continuous and categorical NRI may have improved our prediction performance for each category risk. Nevertheless, as previously stated, critical issues about the limitation of using NRI, either as a continuous or categorical variable, are still under debate and not conclusively solved. In clinical settings, additional stratification strategies for CAD, such as biomarkers, imaging modalities, or genetic profiles, will have to be calculated individually or for the intermediate risk group.

## Conclusions

Overall, our findings demonstrate that GRS is independent of TRF in a single-center cohort with adequate population stratification. GRS improved the predictive risk of FRS across all subgroups in 10-year risk estimates according to the Framingham risk prediction function. A marginal increase in reclassifications measures was found, with a similar pattern across all subgroups. Further debate is warranted about the incorporation of genetic data in subgroups known to be subestimated with respect to standard stratification.

## Supplementary material

The following online material is available for this article:

Table S1 - List of the 32 genetic variants previously associated with CAD risk, selected to create the GRS in our Population.

Table S2 - Reclassification table comparing predicted CAD risk with and without GRS.
