# Supplementary material for: Additional value of a combined genetic risk score to standard cardiovascular stratification
Source: Genet Mol Biol. 2018 Oct-Dec;41(4):766–74. doi: 10.1590/1678-4685-GMB-2017-0173 (PMC6415604; doi:10.1590/1678-4685-GMB-2017-0173)
Supplement: Supplementary file 1 [file 1415-4757-GMB-41-04-0173-s001.pdf]

Supplementary to "Additional value of a combined genetic risk score to standard cardiovascular stratification"

Table S1 – List of the 32 genetic variants previously associated with CAD risk, selected to create the GRS in our Population.

SNP ID	Nearest gene	Chr	Position	Genotypic OR (95%CI)	p value	Allelic OR (95%CI)	p value	MAF (%)	Potential mechanism of action
rs1333049	9p21.3	9	22125504	1.147 (1.036-1.270)$^{+}$	0.008	1.155 (1.041-1.282)	0.007	45.8	Cellular
rs4977574	CDKN2B	9	22098575	1.161 (1.049-1.286)$^{+}$	0.004	1.172 (1.056-1.302)	0.003	42.0	Cellular
rs618675	GJA4	1	34922761	1.143 (0.792-1.649)*	0.475	1.046 (0.918-1.191)	0.502	19.6	Cellular
rs17228212	SMAD3	15	65245693	1.202 (0.888-1.629)*	0.234	1.025 (0.910-1.155)	0.684	25.3	Cellular
rs17465637	MIA3	1	222650187	1.088 (0.971-1.220)$^{+}$	0.148	1.088 (0.971-1.220)	0.147	28.6	Cellular
rs12190287	TCF21	6	134256218	1.230 (1.100-1.375)$^{+}$	<0.0001	1.226 (1.098-1.368)	0.0003	32.7	Cellular
rs3825807	ADAMTS7	15	76876166	1.073- (0.967-1.191)$^{+}$	0.185	1.074 (0.967-1.194)	0.181	41.2	Cellular
rs11556924	ZC3HC1	7	130023656	1.227 (1.058-1.423)*	0.007	1.157 (1.037-1.290)	0.009	34.3	Cellular
rs1332844	PHACTR1	6	12927312	1.113 (1.003-1.235)$^{+}$	0.044	1.113 (1.003-1.236)	0.043	44.3	Cellular
rs2114580	PCSK9	1	55167236	1.079 (0.821-1.417)*	0.587	0.974 (0.866-1.096)	0.665	26.3	Lipids
rs20455	KIF6	6	39357302	1.129 (0.896-1.424)*	0.306	1.060 (0.949-1.184)	0.302	32.8	Lipids
rs7412/ rs429358[1]	APOE[1]	19	44908822/ 44908684	1.261 (1.062-1.497)$^{\#}$	0.008	1.231 (1.056-1.435)	0.008	13.4	Lipids
rs964184	ZNF259	11	116778201	1.131 (0.986-1.298)$^{+}$	0.078	1.130 (0.986-1.295)	0.079	17.7	Lipids
rs599839	PSRC1	1	109279544	1.059 (0.933-1.203)$^{+}$	0.375	1.058 (0.933-1.201)	0.379	21.4	Lipids
rs1801133	MTHFR 677	1	11796321	1.178 (1.017-1.365)$^{\#}$	0.029	1.114 (0.998-1.243)	0.055	33.5	Oxidation
rs1801131	MTHFR 1298	1	11794419	0.944 (0.816-1.093)$^{\#}$	0.443	0.958 (0.854-1.075)	0.465	28.0	Oxidation

SNP ID	Nearest gene	Chr	Position	Genotypic OR (95%CI)	p value	Allelic OR (95%CI)	p value	MAF (%)	Potential mechanism of action
rs705379	PON -108	7	96324583	1.135 (0.950-1.355)#	0.163	1.068 (0.962-1.184)	0.217	46.4	Oxidation
rs662	PON 192	7	95308134	0.836 (0.652-1.072)*	0.157	0.927 (0.828-1.037)	0.186	30.1	Oxidation
rs854560	PON 55	7	95316772	1.161 (1.044-1.290)+	0.006	1.161 (1.044-1.290)	0.006	40.4	Oxidation
rs6922269	MTHFD1L	6	150931849	1.067 (0.804-1.416)*	0.653	0.996 (0.887-1.118)	0.943	27.3	Oxidation
rs5186	AT1R	3	148742201	1.245 (0.906-1.710)*	0.177	1.062 (0.942-1.198)	0.323	24.7	RAS
rs699	AGT	1	230710048	0.932 (0.798-1.090)#	0.380	0.969 (0.873-1.076)	0.552	42.9	RAS
rs4340	ACE	17	61565892	1.165 (1.001-1.355)*	0.048	1.083 (0.973-1.205)	0.143	38.1	RAS
rs4402960	IGF2BP2	3	185793899	1.124 (0.876-1.443)*	0.358	1.020 (0.911-1.141)	0.736	30.8	Diab/Obes
rs1326634	SLC30A8	8	117172544	1.213 (0.914-1.609)#	0.181	1.081 (0.961-1.217)	0.195	25.8	Diab/Obes
rs266729	ADIPOQ	3	186841685	1.209 (1.041-1.403)#	0.013	1.165 (1.030-1.318)	0.015	23.3	Diab/Obes
rs7903146	TCF7L2	10	112998590	0.961 (0.862-1.072)+	0.480	0.962 (0.863-1.072)	0.482	35.3	Diab/Obes
rs17782313	MC4R	18	60183864	1.314 (0.931-1.855)*	0.120	1.016 (0.896-1.152)	0.806	21.6	Diab/Obes
rs1801282	PPARG	3	12351626	1.427 (0.717-2.843)#	0.309	1.164 (0.970-1.396)	0.102	8.8	Diab/Obes
rs1884613	HNF4A	20	44351775	1.159 (0.987-1.360)#	0.072	1.106 (0.960-1.273)	0.163	16.2	Diab/Obes
rs8050136	FTO	16	53782363	1.194 (1.026-1.390)#	0.022	1.129 (1.016-1.255)	0.025	39.7	Diab/Obes
rs1376251	TAS2R 50	12	11030119	1.556 (0.767-3.155)*	0.217	1.080 (0.920-1.267)	0.349	11.9	Diab/Obes

Legend: SNP – Single Nucleotide Polymorphism; Chr – Chromosome; OR – Odds Ratio; CI – Confidence Interval; MAF – Minor Allele Frequency; RAS – Renin-Angiotensin System; Diab/Obes – Diabetes/Obesity; +Additive model;*Recessive model; #Dominant model; [1]Resulting from a Haplotype; Table shows susceptibility loci for CAD, genotypic and allelic ORs and p values for the lead SNP within each locus from GWAS and candidate gene studies. Genotypic ORs are given for additive, recessive or dominant model. Potential mechanism of action is on the basis of what is already known about the function of the nearby genes. It includes “Cellular” (genes associated to cell cycle, cellular migration and inflammation); “Oxidation” (genes involved in pro-oxidative status) and associated with modifiable risk factors such as “Lipids” metabolism, hypertension (“RAS”) and Diabetes/Obesity.
