# Supplementary material for: Additional value of a combined genetic risk score to standard cardiovascular stratification
Source: Genet Mol Biol. 2018 Oct-Dec;41(4):766–74. doi: 10.1590/1678-4685-GMB-2017-0173 (PMC6415604; doi:10.1590/1678-4685-GMB-2017-0173)
Supplement: Supplementary file 2 [file 1415-4757-GMB-41-04-0173-s002.pdf]

Supplementary to "Additional value of a combined genetic risk score to standard cardiovascular stratification"

Table S2 – Reclassification table comparing predicted CAD risk with and without GRS.

Predicted risk (without GRS)	Reclassified predicted risk (with GRS)				% Increase	% Decrease
			**FRS <10%**			
**CAD patients (n=629)**	<25%	25%-50%	50%-75%	75%-100%		
<25%	13◊	4*	0	0	0.3%	0%
25%-50%	41•	105◊	16*	0	1%	2.6%
50%-75%	0	50•	220◊	35*	2.2%	3.2%
75%-100%	0	0	13•	132◊	0%	0.8%
NRI CAD patients			***-7.8%***			
**Healthy controls (n=683)**						
<25%	83◊	16*	0	0	1.2%	0%
25%-50%	157	147◊	12*	0	0.9%	11.9%
50%-75%	0	63•	167◊	9*	0.7%	4.8%
75%-100%	0	0	5•	24◊	0%	0.4%
NRI controls			***27.5%***			
NRI total			***19.7%***			
			**10%≤FRS≤20%**			
**CAD patients (n=583)**	<25%	25%-50%	50%-75%	75%-100%	**% Increase**	**% Decrease**
<25%	0	0	0	0	0%	0%
25%-50%	3•	110◊	44*	0	2.8%	0.2%
50%-75%	0	41•	218◊	105*	6.7%	2.6%
75%-100%	0	0	0	62◊	0%	0%
NRI CAD patients			***18%***			
**Healthy controls (n=466)**						
<25%	2◊	0	0	0	0%	0%
25%-50%	29•	203◊	38*	0	2.9%	2.2%
50%-75%	0	39•	118◊	27*	2%	3%
75%-100%	0	0	0	10◊	0%	0%
NRI controls			***0.6%***			
NRI total			***18.7%***			
			**FRS> 20%**			
**CAD patients (n=354)**	<25%	25%-50%	50%-75%	75%-100%	**% Increase**	**% Decrease**
<25%	0	0	0	0	0%	0%
25%-50%	0	26◊	0	0	0%	0%
50%-75%	0	9•	162◊	32*	2%	0.6%
75%-100%	0	0	2•	123◊	0%	0.1%

Predicted risk (without GRS)	Reclassified predicted risk (with GRS)				% Increase	% Decrease
NRI CAD patients			***5.9%***			
**Healthy controls (n=173)**						
<25%	0	0	0	0	0%	0%
25%-50%	0	46◊	0	0	0%	0%
50%-75%	0	9•	85◊	12*	0.9%	0.7%
75%-100%	0	0	0	21◊	0%	0%
NRI controls			***-1.7%***			
NRI total			***4.2%***			

CAD, Coronary Artery Disease; NRI, Net Reclassification Improvement (categorical NRI); CI, Confidence Interval; GRS, Genetic risk score; FRS, 10-year Framingham risk score. *Number of Individuals who were reclassified into higher risk; •Number of individuals who were reclassified into lower risk; ◊Number of individuals that remained in the same risk group.
